# A retrospective analysis of discharge summaries from a tertiary care hospital medical oncology unit: To assess compliance with documentation of recommended discharge summary components

**DOI:** 10.1002/cnr2.1457

**Published:** 2021-06-21

**Authors:** Jingwei Ge, Alison Davis, Ankit Jain

**Affiliations:** ^1^ ANU Medical School Australian National University Canberra Australia; ^2^ Department of Medical Oncology the Canberra Hospital Garran Australia

**Keywords:** interdisciplinary communication, medical documentation, patient discharge

## Abstract

**Background:**

Discharge summaries are essential for health transition between inpatient hospital teams and outpatient general practices. The patient's outcome is dependent on the quality and timeliness of discharge summaries.

**Aim:**

A retrospective analysis was carried out to assess the compliance with recommended documentation of 697 electronic discharge summaries (eDSs) of oncology inpatients discharged in 2018 from the Canberra Hospital according to the National Guidelines of On‐Screen Presentation of Discharge Summaries.

**Methods and results:**

Individual medical records were identified and screened for the recommended eDS components according to the National Guidelines. Out of the 17 recommended components, nine components were included in all discharge summaries, two components in more than 99% and two components in 95–96% of discharge summaries. The most frequently omitted components include “information provided to the patient,” “ceased medicine” and “procedures,” and these were omitted in 8, 38 and 82% of discharge summaries, respectively.

**Conclusion:**

Overall, most discharge summaries adhered to the national guidelines quite well by including most of the recommended components. However, the discharge summary quality is still inadequate in some domains.

## 1 INTRODUCTION

A hospital discharge summary is a synopsis of information regarding events occurring during the in‐patient care of a patient by a provider or organization. The document is issued after the patient leaves the hospital and is usually issued to the patient's primary carer/general practitioner (GP), in the form of an electronic discharge summary (eDS). It forms the primary method of communication between secondary/tertiary level medical care and primary care.^1^ Studies have shown that high‐quality discharge summaries are pivotal in ensuring patient safety during clinical handover between care settings.^2^, ^3^ In addition, it has also been established that patients can be adversely affected because of delayed, incomplete discharge summaries or discharge summaries carrying incorrect information, leading to increased risk of rehospitalization, complications due to medication error, morbidity and mortality.^4^, ^5^

In 2012, the Australian Commission on Safety and Quality in Health Care (ACSQHC) was appointed to develop and manage a clinical safety program for the My Health Record system. Following the fourth clinical safety review, the Commission identified issues in the presentation of eDSs, including an inconsistent display of information between clinical settings, inconsistent view of terminology between the hospital and the GP software view, and varying format of medications information across hospital discharge summary templates.^1^ To improve the on‐screen presentation of discharge summaries and promote the safety and quality of patient care, the Commission developed a common presentation format for discharge summaries. The National Guidelines for the on‐screen presentation of discharge summaries was first published in 2016 then revised and presented to states and territories on October 1, 2017 The guidelines aim to drive standardization in the way discharge summaries are presented. It provides a detailed structure and format of discharge summaries, including 17 discharge summary components and what should be included in each component.^1^

Previous studies have assessed the quality of discharge summaries based on the presence of key elements. A study in the United States examined adherence of discharge summaries to the U.S. Joint Commission mandated discharge summary components and found that most discharge summaries adequately met most of the Joint Commission standards.^6^ A more recent pilot study in Australia developed a discharge summary assessment tool by identifying components that Australian GPs believed to be important for patient safety in clinical handover.^7^ An Austrian retrospective analysis examined the quality of randomly selected discharge summaries and identified quality issues including the absence of important items and frequent use of unexplained abbreviations.^8^

Although it has been more than 2 years since the publication of the National Guidelines, to the best of our knowledge, no studies have examined how well discharge summaries adhere to the Australian National Guidelines in any specific sub‐speciality. Therefore, our study aimed to perform a retrospective audit to assess the completeness of the provision of the 17 mandatory components according to the National Guidelines and the timeliness of providing the discharge summaries in a large inpatient oncology unit.

## 2 METHODS

### 2.1 Primary and secondary objectives of the study

The primary objective of our study is to determine whether the discharge summaries of oncology patients in Canberra hospital comply with the national guidelines for on‐screen presentation of discharge summaries in presenting all mandated components specified in the National Guidelines.

The secondary objective is to assess the timeliness of providing the discharge summaries.

### 2.2 Sample

**FIGURE 1:**
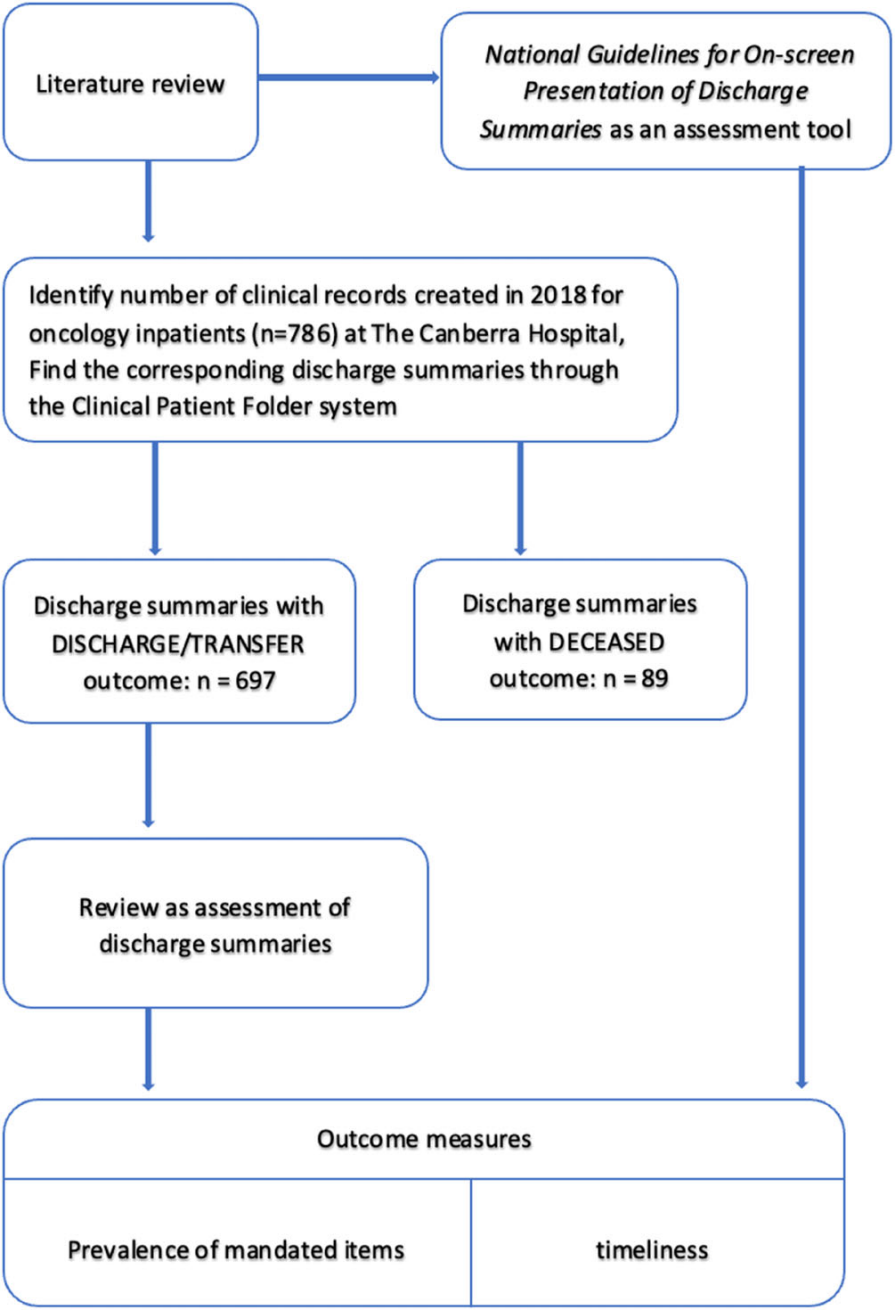
Study flow chart

We identified medical records (*N* = 786) for patients who were admitted to the medical oncology unit between January 1, 2018 and December 31, 2018 at The Canberra Hospital. Each hospitalization was treated as a separate event and the multiple discharge summaries for the same patient were assessed separately. We excluded the discharge summaries of 89 patients who were deceased during the admission. In the end total of 697 discharge summaries were analysed. (Figure 1).

The study was approved by the Australian Capital Territory (ACT) Health Research Ethics Low Risk Sub‐Committee.

### 2.3 Data collection

A list of in‐patient oncology admissions during the specified period was obtained from the medical record department of the Canberra Hospital, with those deceased during admission excluded. Individual medical records were retrieved online from the clinical portal and clinical patient folder system and the discharge summary for each admission was examined according to the National Guidelines.

The 17 mandatory components specified by the National Guidelines include: (a) patient details, (b) hospital details, (c) recipients, (d) author, (e) presentation details, (f) problems and diagnoses, (g) procedures, (h) clinical summary, (i) allergies/adverse reactions, (j) medicines on discharge, (k) ceased medicines, (l) alerts, (m) recommendations, (n) follow‐up appointments, (o) information provided to the patient, (p) recipients and (q) selected investigation results.

We screened each discharge summary against the 17 mandatory components. Each component was recorded as either PRESENT or ABSENT. For instance, for the “allergies/adverse reactions” component, if any allergies or no known drug allergies (NKDA) or nil were documented, PRESENT would be recorded for this component. On the other hand, if the component was left blank, with or without the component heading, it would be recorded as ABSENT.

In addition, we collected time from patient discharge to completion of the document for each discharge summary.

### 2.4 Statistical analysis

All data extracted from the discharge summaries were anonymised and collated without individual patient identifiers to maintain confidentiality. The prevalence of each component was calculated as a percentage, the average prevalence for all components was also calculated.

To assess the timeliness of providing the discharge summaries, we calculate the percent of discharge summaries that were completed (a) on the day of discharge, (b) more than 48 hours after discharge and (c) more than a week after discharge.

## 3 RESULTS

Out of the 17 components, nine components were included in all discharge summaries. These components are “patient details,” “hospital details,” “recipients,” “author,” “presentation details,” “problems and diagnosis,” “clinical summary,” “recipients' details” and “selected investigation results.” The “medicines on discharge” and “recommendations” components were included in virtually all discharge summaries (99 to 100%). The “allergies/adverse reactions” and “follow‐up appointments” components were included in the vast majority of discharge summaries (95–97%; Table 1).

**TABLE 1:** Prevalence of National Guidelines recommended components

National Guidelines recommended components	Prevalence (%)
A) Patient details	100
B) Hospital details	100
C) Recipients	100
D) Author	100
E) Presentation details	100
F) Problems and diagnosis	100
G) Procedures	18
H) Clinical summary	100
I) Allergies/adverse reactions	96
J) Medicines on discharge	99
K) Ceased medicine	62
L) Alerts	0
M) Recommendations	99
N) Follow‐up appointments	95
O) Information provided to the patient	92
P) Recipients' details	100
Q) Selected investigation results	100

Other components were included less frequently, with the “information provided to the patient” component included in 92%, “ceased medicine” in 62% and the “procedures” component included in only 18% of discharge summaries (Table 1).

We could not identify a component in the discharge summaries that corresponded with the “alert” component recommended by the National Guidelines. Therefore, this component was considered absent in all discharge summaries (Table 1).

In terms of timeliness, 82% of discharge summaries were completed on the day of discharge, 7% were completed in 1 to 2 working days after discharge, 4% were completed in 2–6 working days after discharge, 7% were completed in more than 7 working days (Table 2).

**TABLE 2:** Timeliness of discharge summaries based on time of completion from patient discharge

Number of working days between discharge and receipt of the summary	Percentage (%)
<1 (on the day of discharge)	82
1–2 days	7
2‐6 days	4
>7 days	7

## 4 DISCUSSION

This study confirms that the discharge summaries of medical oncology inpatients at our institution are, in general, largely compliant with the National Guidelines, with 13 of the 17 recommended components present in 95% of summaries. These findings were largely consistent with previous studies, which showed good completion rates of the majority of discharge summary components.^6^, ^7^

In 2008, Kind and Smith performed a similar audit to examine the completeness of discharge summary documentation by assessing the prevalence of the six Joint‐commission mandated components, including “reason for hospitalization,” “significant findings,” “procedures and treatment provided,” “patient's discharge conditions,” “patient and family instructions” and “attending physician's signature.”^6^ In terms of similarities, the previous study found that most discharge summaries adequately meet most of the Joint Commission standards, which is consistent with our findings that most discharge summaries adequately adhered to the National Guidelines. In terms of differences, our study showed more frequent omission of certain components. In the previous audit, the most frequently omitted component was “patient's discharge condition” and it was still included in 79–90% of discharge summaries. In our study, the “ceased medicine” and “procedures” component showed lower prevalence and was only found in only 62 and 18% of discharge summaries respectively. However, this does not necessarily indicate a poorer quality from our sample. Firstly, the number of items required to be documented is less in the previous study as the joint commission mandated six components while the National Guidelines specified 17 components. Therefore, the practitioner can meet the standards with minimal documentation since there were fewer components to be completed. Some clinically significant information, for instance, allergy and adverse reactions, were not mandated by the Joint Commission. In addition, the high rate of adherence to the Joint Commission components is likely since the definition for these components is extremely broad.^6^ For instance, the Joint Commission mandated component “patient/family instruction” was defined as discharge medications activity orders AND/OR therapy orders AND/OR dietary instructions AND/OR plans for medical follow‐up. In this case, satisfying one of these smaller umbrella terms for “patient/family instruction” would satisfy the requirement for this component. However, this single component covers the National Guidelines recommended components “medicines on discharge,” “ceased medicine,” “follow‐up appointments” and “selected investigation results.” As each of these components was assessed individually, the omission of any of these components would be documented and reflected by the prevalence.

In 2017, Mahfouz et al. developed an Australian discharge summary quality assessment tool by identifying components that Australian GPs believed as being most important.^7^ The five most important items being identified include “list of medications on discharge,” “reason for admission,” “treatment in hospital,” “details of follow‐up arrangements” and “list of diagnoses on discharge.”^7^ These correspond to “medicines on discharge,” “presentation details,” “clinical summary,” “follow‐up appointments” and “problems and diagnosis” components specified by the National Guidelines. Table 3 listed the five most important discharge summary components rated by Australian GPs from Mahfouz et al.'s study and the prevalence of these components in our study. We found that the five most important items were included in the vast majority (95–100%) of discharge summaries from our sample, with three components, “reason for admission,” “treatment in hospital” and “list of diagnoses on discharge” being included in all discharge summaries. In reality, it is unlikely for all discharge summaries to include all the recommended components. But at least it is essential to make sure the most important information is included in the discharge summaries to maximally avoid patient harm. Our study showed good completion rates of the established important components, which is indicative of good discharge summary quality.

**TABLE 3:** Prevalence of components identified as most important by Australian General Practitioners

Rank	Important discharge summary items identified by Australian GPs	Corresponding components in the National Guidelines	Prevalence (%)
1	List of medications on discharge	Medicines on discharge	99
2	Reason for admission	Presentation details	100
3	Treatment in hospital	Clinical summary	100
4	Details of follow‐up arrangements	Follow‐up appointments	95
5	List of diagnoses on discharge	Problems and diagnosis	100

We found that 82% of discharge summaries were received within the day of discharge, 93% were received within a week and 97% were received within 2 weeks. This compares favorably with other studies, where the percentage of discharge summaries received within the day of discharge ranged between 26 and 55%.^9^, ^10^ Despite the difference in the proportion of discharge summaries delivered in a timely fashion, all studies identified delays in delivery. Improvements in the percentage of timely completion and delivery of discharge summaries noted in our study could be due to the implementation of eDSs and the standardised format.^11^ The harmful effects of delayed information transfer and its relation to readmission and poor outcomes have been well established.^3^, ^4^, ^5^ A linear trend was observed between the delay in transmission of discharge summary and the readmission rate of the patient.^12^ Ideally, discharge summaries should be made available to the primary care providers on the day of discharge.

### 4.1 Causes of frequent omissions and interventions to improve quality

1. The practitioner did not record any information, potentially due to the presumption that leaving a component blank means the information was not applicable:

The component “ceased medicine” was present in 62% of discharge summaries. Although not all patients would have alterations to medications during hospitalization, this information should be documented under the appropriate section as N/A or nil if it was not available, rather than leaving the component blank. From a reader's point of view, it would be difficult to recognize whether there was no change to medications or the practitioner has omitted this information. Uninformed medication change is associated with increased emergency department visits, readmissions, disabilities and even death, which is an ameliorable medical error that should be avoided.^13^

2. This information was recorded under other components of the discharge summaries:

The component “procedures” was found in only 17% of the discharge summaries. Understandably, not all patients would have certain operations done. However, apart from the presumption that has been discussed, the lack of structural stringency is another reason for omitting these components. Some discharge summaries incorporate information related to operations in the “clinical summary” component and did not have a separate “procedure” component. However, as required by the National Guidelines, it is important to have a separate section for this component as it would be easier and more straightforward for the reader of this discharge summary so that they can obtain useful information in the first place.

3. The template provided by the hospital does not include certain components

The “alert” component was not present in all discharge summaries due to the template adopted by the Canberra Hospital. According to the National Guidelines, the content that should be presented in the “alert” component include a list of alerts that may affect the patient's continuity of care. The definition itself was not very clear. Also, the National Guidelines noted that using this section is at the discretion of the author and it is not automatically populated. Therefore, it should not be concerning if this particular component is missing in discharge summaries.

### 4.2 Limitations and future research directions

The primary limitation of the study is related to the generalizability of our results. The results are solely based on data collected from a single speciality within a single institution, and therefore may differ between specialities and institutions. Still medical oncology speciality has a broad range of problems and various subspecialities can still learn from this research. As addressed earlier, the fact that the component “procedures” were frequently omitted from the discharge summaries may be a function of the fact that those patients did not have any procedures done during admission, likewise for “ceased medicine.” Given that, in specialities where patients are more likely to receive operational procedures, for instance, the surgical speciality, it is expected that the compliance rate to the component “procedures” would be higher and in specialities where patients are less likely to receive operational procedures, the compliance rate is expected to be lower. However, for other components that are more universal to all patients, for instance “presentation details,” the compliance rate is expected to be more stable across different disciplines. Also, since this work is solely based on The Canberra Hospital, it is unclear whether the results can be replicated by other institutions or health care facilities since each facility adopts its own medical record template and training system for the authors of the medical records. There is no comparative arm to know what was the quality of discharge summaries before the release of national guidelines for on‐screen presentation of discharge summaries by ACSQHC in 2017. It will be also important to do a mixed method analysis with a survey of various subspeciality physicians to understand what component of mandated discharge summary components works and which do not work.

Despite the limited generalizability, some insights could still be gained from our results. The first one being the necessity of creating a universal electronic medical record template which is based on The National Guidelines. If this is not possible, each healthcare facility should modify its own electronic medical record template to make sure it is in accordance with The National Guidelines. Secondly, training provided to the authors of the medical records should be consistent with The National Guidelines. For instance, when there are no procedures done or no medication provided, authors should record this information as “not available” on the electronic medical record.

There is a lack of a universal definition for the “quality” of discharge summaries since a discharge summary can be assessed by multiple indicators. For instance, the adequacy of the discharge summary components in addition to the mere presence of the components should be taken into consideration when assessing quality.^14^ Yemm et al. suggested in a survey that the key characteristics of a discharge summary include accuracy, completeness, timeliness and grammar.^2^ In our study, we have only assessed completeness according to the National Guidelines and timeliness, leaving the firstly ranked characteristic “accuracy” unexamined. The National Guidelines included recommendations on the “position,” “heading,” “format” and “content” of each specific component. However, due to the limited resources we had, we were not able to look into those details. Therefore, we were unable to accurately assess the quality or accuracy of the discharge summaries since some components may be present but poorly or incorrectly documented. Future research should explore not only whether the discharge summaries adhered to the National Guidelines but also the accuracy of the content of each component.

There lacks a universal standard for how soon discharge summaries should be made available to primary care providers. Ideally, discharge summaries should be completed and delivered on the day of discharge, but at least before the first follow‐up appointment. Previous studies have collected data on the availability of discharge summaries at the post‐discharge visit, which is a more accurate indicator of timeliness than the number of days between discharge and receipt of the summary.^3^ In our study, we were not able to do this since despite the 91% prevalence of the “details of follow‐up arrangements” component, in many discharge summaries the exact date of follow‐up appointments were not documented. Therefore, we were unable to calculate the percentage of discharge summaries that were made available by the first GP visit. The factors which may impact the completion of discharge summary in a timely manner are after‐hours discharge and discharge done on weekends. It will be good to look into these factors in future studies.

We did not collect data on author details. It has been noted that the task of writing a discharge summary usually falls on interns, and this could be problematic since the interns are less experienced and they are usually not the medical staff team member who knows the patient best.^15^ Future studies can look into the proportion of discharge summaries completed by interns and assess if there is any discrepancy in the quality of discharge summaries completed by authors of different levels of clinical experience.

Although it has been established that delayed or incomplete discharge summaries can lead to adverse events during the patient transition. In our study, we were unable to validate whether there is any correlation between delayed or incomplete discharge summary and the development of complications. Future studies can collect data on patient outcomes after discharge and analysis if there exists a correlation between discharge summary quality and patient safety.

## 5 CONCLUSION

In conclusion, most discharge summaries completed for medical oncology inpatients adhered well to the national guidelines by including most of the recommended components. However, there were several domains where compliance was suboptimal, namely “alert,” “procedures,” “ceased medicine” and “information provided to the patient.” Most discharge summaries were completed on the day of discharge and the vast majority within a week of discharge. Timely delivery of a high‐quality discharge summary is pivotal in ensuring patient safety. Delays and omissions should be avoided to reduce patient harm.

## CONFLICT OF INTEREST

The authors declared there is no conflict of interest.

## ETHICAL STATEMENT

The study was approved by the institutional review board, patient consent was not required as it was a retrospective audit.

## AUTHOR CONTRIBUTIONS

*Conception and Design*, A.J.; *Data Collection, Analysis and Interpretation*, J.G.; *Interpretation*, A.J. and A.D.; *Manuscript Writing*, J.G. and A.J.; *Manuscript Review*, J.G., A.J., and A.D.

## Data Availability

Research data are not shared.
